# Care-seeking patterns and timely access to care among survivors of sexual violence in North Kivu, the Democratic Republic of the Congo: a retrospective file-based study

**DOI:** 10.1080/16549716.2024.2336708

**Published:** 2024-04-25

**Authors:** Hanna Reinholdz, Anette Agardh, Meggy Verputten, Joseph Byenda, Helena Frielingsdorf

**Affiliations:** a Department of Clinical Sciences, Division of Social Medicine and Global Health, Lund University, Malmö, Sweden; b Public Health Department, Médecins Sans Frontières, Amsterdam, Netherlands; c North Kivu Country Programme, Médecins Sans Frontières, Goma, The Democratic Republic of the Congo; d Department of Health, Medicine and Caring Sciences, Division of Society and Health, Linköping University, Linköping, Sweden

**Keywords:** Gender-based violence, sexual abuse, Sexual assault, rape, help-seeking behaviour, Democratic Republic of the Congo

## Abstract

**Background:**

Sexual violence is widespread in war-torn North Kivu province in the Democratic Republic of the Congo (DRC). Timely access to care is crucial for the healing and wellbeing of survivors of sexual violence, but is problematic due to a variety of barriers. Through a better understanding of care-seeking behaviours and factors influencing timely access to care, programmes can be adapted to overcome some of the barriers faced by survivors of sexual violence.

**Objective:**

The aim of this study was to describe demographics, care-seeking patterns and factors influencing timely care-seeking by survivors of sexual violence.

**Methods:**

Retrospective file-based data analysis of sexual violence survivors accessing care within two Médecins Sans Frontières (MSF) programmes supporting the Ministry of Health, in North Kivu, DRC, 2014–2018.

**Results:**

Most survivors (66%) sought care at specialised sexual violence clinics and a majority of the survivors were self-referred (51%). Most survivors seeking care (70%) did so within 3 days. Male survivors accessing care were significantly more likely to seek care within 3 days compared to females. All age groups under 50 years old were more likely to seek care within 3 days compared to those aged 50 years and older. Being referred by the community, a family member, mobile clinic or authorities was significantly associated with less probability of seeking care within 3 days compared to being self-referred.

**Conclusion:**

Access to timely health care for survivors of sexual violence in North Kivu, DRC, is challenging and varies between different groups of survivors. Providers responding to survivors of sexual violence need to adapt models of care and awareness raising strategies to ensure that programmes are developed to enable timely access to care for all survivors. More research is needed to further understand the barriers and enablers to access timely care for different groups of survivors.

## Paper Context

- **Main findings:** Timely access to care for survivors of sexual violence is crucial yet challenging in many places, including in North Kivu, the Democratic Republic of the Congo. This study shows that a majority of survivors access care through specialised clinics, that access is limited for male and child survivors, and highlights factors influencing timely access to care for survivors of sexual violence.
- **Added knowledge:** This study shows that age, sex, and different referral pathways impact timely care seeking among survivors of sexual violence accessing care.
- **Global health impact for policy and action:** A better understanding of care-seeking patterns and which factors influence timely care seeking is useful when designing and implementing programmes responding to survivors of sexual violence.

## Introduction

For survivors of sexual violence, timely access to medical care and psychosocial support is crucial for healing and recovery. Yet, most survivors of sexual violence never seek health care [1] and the ones who do seek health care do not always manage to do so within the three-day window to be able to receive the full package of care [2,3]. In North Kivu, in eastern Democratic Republic of the Congo (DRC), sexual violence is pervasive and access to health care is limited. Prevalence estimates from the World Health Organization (WHO) indicate that 47% of women and girls above 15 years in DRC have experienced physical and/or sexual violence by an intimate partner and that 6% have experienced non-partner sexual violence in their lifetime [4]. Another study shows that around 40% of women and 24% of men in eastern DRC have experienced sexual violence and among those, 74% of the women and 65% of the men reported experience of conflict-related sexual violence [5]. The conflict-related sexual violence in DRC has been described as widespread, deliberate, strategic and systematic, with numerous reports of mass rapes as well as rapes with deliberate torture and extreme violence [6–8].

Survivors of sexual violence are at risk for several medical and psychological sequelae as well as negative social consequences [9–15]. Medical care for survivors of sexual violence in humanitarian settings includes care for wounds and injuries, post-exposure prophylaxis (PEP) of STIs, including human immunodeficiency virus (HIV) and hepatitis B, emergency contraception and management of unwanted pregnancies to the full extent of the law [16,17]. As part of quality response, survivors should also be offered psychological support and access to protection and social and legal support services [16,18]. As the HIV PEP is only effective if it is started within 72 hours of the incident and emergency contraception only effective if taken within 120 hours [17], timely access to care is crucial.

The proportion of survivors accessing care within 3 days in eastern DRC varies considerably between different studies, ranging between 1% and 86% [2,3,13,19–21]. Different barriers to seeking or accessing health care after a sexual violence incident in DRC are lack of money for transport, fear of disclosure, shame, social isolation, stigmatisation by community, fear that service providers will not be sensitive, lack of knowledge of the services available and poor quality or accessibility of the services [2,9,22].

Médecins Sans Frontières (MSF) is, in collaboration with the Ministry of Health, offering comprehensive care to survivors of sexual violence through different models of care in DRC. However, access to care still seems to be limited and in order to improve timely access to care for survivors of sexual violence, it is important to understand the current care-seeking patterns. With better understanding of the care-seeking patterns, models of care, awareness raising strategies and referral pathways can be appropriately adapted. Therefore, this study aimed to describe demographic background and care-seeking patterns as well as to assess factors that influence timely access to care among survivors of sexual violence in MSF programmes in North Kivu, DRC. More specifically, the study sought to examine the age, sex, referral patterns, type of healthcare facility where survivors accessed care, timing of care-seeking among sexual violence survivors and the association between timely access to care and age, sex and referral pattern.

## Methods

### Study design

This was a retrospective file-based study, using routinely collected data from MSF programmes for survivors of sexual violence in Walikale and Mweso, North Kivu, DRC, recorded between 1 January 2014 and 31 December 2018.

### Setting

The study took place in the province of North Kivu located in Eastern DRC, an area affected by protracted conflict and displacements. DRC has a population of almost 96 million people [23] and around 6 million of those live in North Kivu (2015 data) [24]. DRC is ranked 179 out of 191 countries and territories on the Humanitarian Development Index and has a poverty rate of 77% [25]. MSF is providing health care in several locations in DRC and creating access to comprehensive care for survivors of sexual violence is one of their activities. In North Kivu, MSF is working in close collaboration with the Ministry of Health and is (among other activities) supporting Ministry of Health run hospitals and primary healthcare facilities in providing care to survivors of sexual violence. MSF is also providing care to survivors of sexual violence through a specialised model of care called the Tumaini clinics (‘hope’ in Swahili) within their programmes in the Walikale and Mweso health zones. The Tumaini clinics are combining care to survivors of sexual violence and all forms of intimate partner violence with treatment for STIs, contraception counselling and provision, safe abortion care and mental health counselling. Integrating sexual violence care with other activities aims to create a safe entry point for survivors without exposure and ensures a well-trained team of medical staff and mental health counsellors. MSF programmes also include awareness raising activities around sexual violence, conducted both by MSF health promotion staff and community health workers who are a part of the national healthcare system.

Data were collected from two MSF programmes in North Kivu; Mweso and Walikale. The two programmes were purposively selected as they are using both the Tumaini model of care and integrated models of care in primary healthcare centres and hospitals, giving an opportunity to examine the care-seeking patterns among survivors accessing care in the different healthcare facilities and sexual violence care models. Data from all patients registered as survivors of sexual violence (including ‘rape’ and ‘sexual violence other than rape’) in the two MSF programmes during 1 January 2014 to 31 December 2018 were included. ‘rape’ was defined as non-consensual penetration of anus, mouth or vagina by a penis, another body part or a foreign object. ‘sexual violence other than rape’ included all types of sexual violence, defined as a sexual act or attempt to obtain a sexual act without consent, excluding rape as defined above (including, for example, attempted rapes and forced masturbation). All accounts of rape and other types of sexual violence are based on self-reporting by the survivors.

### Data collection

Data were collected as part of the routine medical activities. Data from individual survivors were registered on standardised medical files by the healthcare worker providing care to the survivor. Key data were transferred from the medical files to a tally sheet by the staff in each healthcare facility. The completed tally sheets were transported to the MSF office in either Mweso or Walikale and shared with the data administrator who entered the data into a password-protected Excel sheet. All data were de-identified and cannot be traced back to any individual. Quality control checks were done by the projects’ medical team leaders on a regular basis, by randomly checking data entered in the data base against medical files or data tally sheets.

#### Sample size

Sample size calculations were made to determine the sample size needed to perform the regression analysis used. The sample size calculation was based on the work of Peduzzi et al. [26] suggesting that *N* = 10k/p where *N* = minimum number of cases to be included, k = number of covariates and *p* = smallest proportions of cases (negative or positive). For the analysis, three covariates were used, k = 3 (sex, age and referral pathway), and p was set to 1% (0.01), yielding *N* = 10 × 3/0.05 = 3000. The p was set as low as 1% to ensure that analyses could be made even if a very small proportion of the population would not be receiving care (based on knowledge from staff working in the projects).

### Variables

Variables included in descriptive analyses were the total number of survivors of sexual violence, sex, age, time between the sexual violence incident and the visit at the healthcare facility, referral pathway and type of healthcare facility visited.

In the logistic regression models, the dependent variable used was time between the incident and the visit at the healthcare facility, categorised as the dichotomous variable seeking care within 3 days (yes or no). The dichotomous variable was classified as ‘yes’ for survivors seeking health care on day 0, 1, 2, and 3 after the incident, even though this might include survivors seeking care up to 96 hours after the incident. This is due to the fact that no information about the exact time of the incident or the visit was available in the dataset but only the day of incident and day of visit.

The independent variables included in the logistic regression models were sex, age and referral pathways.

Sex was used as a dichotomous variable; female or male.

Age was recorded as a continuous measure categorised as groups; 0–9 years, 10–19 years, 20–29 years, 30–39 years, 40–49 years and ≥50 years to provide a separate group for adolescents as defined by WHO [27] and to create equal intervals.

Referral pathway was categorised according to how the survivors were referred to the healthcare facility: self-referred, health promotion team, community health workers, community, psychosocial staff, family member, mobile clinic, local authorities, partner or other.

### Analysis

Bivariable and multivariable logistic regression models were used to examine the association between age, sex and referral pathway and the outcome of seeking care within 3 days of the sexual violence incident. Multivariable logistic regression models were used to adjust for age and sex. The results are shown as odds ratios (ORs) and 95% CIs. Two-tailed *p*-values < 0.05 were considered significant.

#### Missing data

Missing clinical data were assumed to be missing completely at random. The missing data in the data set varied between 0% and 0.5% for the different variables. Complete-case analysis was used since a minimal bias was believed to occur due to the low rate of missing cases [28].

## Results

Total number of sexual violence survivors seeking care was 4684; among those 4655 reported being raped and 28 reported experience of sexual violence other than rape. There were 4146 survivors accessing care in the Mweso programme and 538 survivors within the Walikale programme. The number of survivors accessing care varied over the years, with the highest number of survivors accessing care in 2017 (Table 1).

**Table 1.** Sex and age group of survivors of sexual violence accessing care in MSF programmes in Mweso and Walikale, DRC, 2014–2018.

		Age							
Year	Sex	0–9	10–19	20–29	30–39	40–49	≥50	Missing data^a^	Total
2014	*Female*	14	195	232	154	66	30		691
	*Male*	0	4	12	7	8	1		32
	*Total*	14	199	244	161	74	31		723
2015	*Female*	24	153	186	144	88	30		625
	*Male*	0	0	11	10	1	0		22
	*Total*	24	153	197	154	89	30		647
2016	*Female*	20	248	309	287	116	52		1032
	*Male*	1	6	8	8	1	1		25
	*Total*	21	254	317	295	117	53		1057
2017	*Female*	25	316	377	291	124	50	1	1184
	*Male*	0	6	8	8	1	2		25
	*Total*	25	322	385	299	125	52	1	1209
2018	*Female*	24	281	402	202	90	20		1019
	*Male*	1	10	9	6	3	0		29
	*Total*	25	291	411	208	93	20		1048
2014–2018	*Female*	107	1193	1506	1078	484	182	1	4551
	*Male*	2	26	48	39	14	4	0	133
	*Total*	109	1219	1554	1117	498	186	1	4684

^a^Data concerning age of survivor missing.

Among all survivors of sexual violence accessing care, 97.2% (*n* = 4551) were women and 2.8% (*n* = 133) were men. The age range was from 1 to 80 years. The mean age was 26.95 ± 11 years and median age 26 years (IQR 18–34). Out of all survivors accessing care, 2.3% (*n* = 109) were 0–9 years old and 26% were adolescents, aged 10–19 years (*n* = 1219). The number of survivors accessing care per year, sex and age group is shown in Table 1. The largest age group, both among female and male survivors, was 20–29 years.

The majority of the survivors seeking care, 66% (*n* = 3081) sought care at a Tumaini clinic; 5% (*n* = 242) sought care at a hospital and 29% (*n* = 1361) at a health centre. The Tumaini clinics were the most commonly accessed type of healthcare facility for both sexes and all age groups. The number of healthcare facilities supported by MSF for sexual violence care provision and the number of survivors accessing care per type of facility, project and year are shown in Table 2. As shown in the table, some facilities were only running or providing care during a part of the year.

**Table 2.** Healthcare facilities offering sexual violence care with MSF support and survivors of sexual violence accessing care in those facilities in Walikale and Mweso, North Kivu, DRC 2014–2018.

	*Number of MSF supported healthcare facilities with sexual violence care provision*				*Number of survivors of sexual violence accessing care*		
Year		Health Centres	Hospitals	Tumaini (specialised sexual violence clinic)	Health Centres	Hospitals	Tumaini (specialised sexual violence clinic)
2014	*Walikale*	3	1	0	24	4	0
	*Mweso*	4	1	2^a^	161	0	534
	*Total*	7	2	1	185	4	534
2015	*Walikale*	3	1	0	26	44	0
	*Mweso*	5^b^	1	1	167	0	410
	*Total*	8	2	1	193	44	410
2016	*Walikale*	4^c^	1	1^d^	45	194	46
	*Mweso*	5	1	2^e^	220	0	552
	*Total*	9	2		265	194	598
2017	*Walikale*	4	1	1	32	0	77
	*Mweso*	5	1	2	293	0	807
	*Total*	9	2	3	325	0	884
2018	*Walikale*	4	1	1	9	0	37
	*Mweso*	11^f^	1	2	384	0	618
	*Total*	15	2	3	393	0	655
2014–2018	*Walikale*				136	242	160
	*Mweso*				1225	0	2921
	*Total*				1361	242	3081

^a^One of them only January to August.

^b^One of them only in December.

^c^One of them only September–December.

^d^Only November–December.

^e^One of them only April–December.

^f^Six of them only March–December.

The most commonly reported referral pathways were being self-referred (50.7%, *n* = 2375), being referred by the health promotion team (MSF staff) (17.3%, *n* = 808) or being referred by someone in the community (8.9% *n* = 415) (Table 3). Self-referral was most common in the age group 20–29 (54.0%). In both the Tumainis and the health centres, being self-referred was the most common referral pathway, whereas in the hospitals the most common referral pathway was through local authorities (Table 3).

**Table 3.** Referral pathways for survivors of sexual violence accessing care in different type of healthcare facilities in MSF programmes in North Kivu, DRC, 2014–2018.

	*Tumaini*		*Health Centre*		*Hospital*		*Total*	
	n	%	n	%	n	%	n	%
*Referral pathway*								
*Self-referral*	1459	47.22%	902	66.77%	14	5.83%	2375	50.74%
*Health promotion team*	803	25.99%	5	0.37%	0	0.00%	808	17.26%
*Community health worker*	195	6.31%	117	8.66%	15	6.25%	327	6.99%
*Community*	366	11.84%	49	3.63%	0	0.00%	415	8.87%
*Psychosocial staff*	76	2.46%	105	7.77%	0	0.00%	181	3.87%
*Family member*	35	1.13%	20	1.48%	29	12.08%	84	1.79%
*Mobile clinic*	3	0.10%	3	0.22%	25	10.42%	31	0.66%
*Local authorities*	71	2.30%	62	4.59%	154	64.17%	287	6.13%
*Partner*	0	0.00%	3	0.22%	2	0.83%	5	0.11%
*Other*	82	2.65%	85	6.29%	1	0.42%	168	3.59%

In the Mweso programme 75.3% of the survivors accessing care sought care within 3 days and in the Walikale programme 26.8% (Table 4). An additional 6.3% sought care between 3 and 5 days after the incident. The percentage of survivors seeking care within 3 days was highest in the health centres, 82.7%, followed by 68.0% in the Tumainis. In the hospitals only 18.8% of survivors sought care within 3 days (Table 4).

**Table 4.** Timing of care seeking among survivors of sexual violence accessing care in different healthcare facilities within MSF programmes in Walikale and Mweso, DRC, 2014–2018.

	Percentage of total survivors accessing care											
Days between sexual violence incident and receiving care	Mweso				Walikale				Total			
	Tumaini	Health Centre	Hospital	Total	Tumaini	Health Centre	Hospital	Total	Tumaini	Health Centre	Hospital	Total
*0–3*	70.55%	86.45%	-	75.25%	23.67%	46.03%	18.83%	26.78%	67.98%	82.68%	18.83%	69.72%
*4–5*	7.61%	3.18%	-	6.30%	1.78%	3.17%	10.46%	5.99%	7.29%	3.18%	10.46%	6.27%
*6–30*	11.04%	6.20%	-	9.61%	13.61%	18.25%	41.00%	26.97%	11.18%	7.33%	41.00%	11.59%
*31–90*	5.76%	2.78%	-	4.88%	15.38%	10.32%	22.18%	17.23%	6.29%	3.48%	22.18%	6.29%
*91–180*	2.57%	0.82%	-	2.05%	8.28%	12.70%	5.44%	8.05%	2.88%	1.92%	5.44%	2.74%
*>180*	2.47%	0.57%	-	1.91%	37.28%	9.52%	2.09%	14.98%	4.37%	1.41%	2.09%	3.40%

Table 5 shows the result of the bivariable logistic regression models for each independent variable as well as the result of the multivariable logistic regression model adjusting for sex, age and referral pathway. All age groups were significantly associated with higher probability of seeking care within 3 days compared to the age group ≥50 years (*p* < 0.001) (Table 5), the highest probability was for the age group 0–9 years. Males had a significantly higher probability of seeking care within 3 days compared to female (*p* < 0.001) (Table 5). Being referred by the community, a family member, a mobile clinic or local authorities was significantly associated with lower probability of seeking care within 3 days compared to being self-referred (p-values < 0.001 for referral by a family member, mobile clinic and local authorities, p-value < 0.005 for referral by community) (Table 5).

**Table 5.** Association between age, sex and referral pathways and access to care within 3 days of a sexual violence incident among survivors accessing care in MSF programmes in Walikale and Mweso, DRC 2014–2018.

	*Bivariable logistic regression*				*Multivariable logistic regression*			
Dependent variable:		95% CI		p-value	Adjusted OR	95% CI		p-value
*Accessing care within 3 days after the sexual violence incident*	OR	lower	upper			lower	upper	
Independent variable:								
***Age***								
*=>50*	*ref*				*ref*			
*0–9*	**2.903**	**1.743**	**4.833**	**<0.001**	**3.375**	**1.931**	**5.897**	**<0.001**
*10–19*	**2.379**	**1.740**	**3.253**	**<0.001**	**2.067**	**1.476**	**2.895**	**<0.001**
*20–29*	**3.310**	**2.426**	**4.515**	**<0.001**	**2.907**	**2.081**	**4.061**	**<0.001**
*30–39*	**2.603**	**1.898**	**3.570**	**<0.001**	**2.213**	**1.576**	**3.107**	**<0.001**
*40–49*	**1.859**	**1.322**	**2.615**	**<0.001**	**1.597**	**1.108**	**2.301**	**0.012**
***Sex***								
*Female*	*ref*				*ref*			
*Male*	**2.846**	**1.725**	**4.696**	**<0.001**	**2.679**	**2.673**	**1.592**	**4.489**
***Referral pathway***								
*Self-referral*	*ref*				*ref*			
*Health promotion team*	1.125	0.934	1.353	0.214	1.169	0.969	1.410	0.104
*Community health worker*	1.071	0.82	1.397	0.615	1.161	0.887	1.520	0.277
*Community*	**0.783**	**0.624**	**0.982**	**0.034**	**0.791**	**0.629**	**0.995**	**0.045**
*Psychosocial staff*	1.022	0.724	1.442	0.903	1.028	0.726	1.456	0.876
*Family member*	**0.282**	**0.182**	**0.438**	**<0.001**	**0.285**	**0.180**	**0.450**	**<0.001**
*Mobile clinic*	**0.086**	**0.035**	**0.211**	**<0.001**	**0.097**	**0.039**	**0.239**	**<0.001**
*Local authorities*	**0.089**	**0.066**	**0.121**	**<0.001**	**0.091**	**0.066**	**0.123**	**<0.001**
*Partner*	0.239	0.04	1.433	0.117	0.243	0.039	1.509	0.129
*Other*	1.477	0.999	2.184	0.051	**1.566**	**1.054**	**2.326**	**0.026**

Hosmer and Lemeshow test for goodness of fit of multiple logistic regression model; p-value 0.595. Bold indicates significant values (p-value < 0.05). OR = Odds Ratio, CI = Confidence Interval.

The associations described above were significant both in the bivariable logistic regression models for each independent variable and in the multivariable logistic regression model including age, sex and referral pattern. According to the Hosmer and Lemeshow test for goodness of fit, the multivariable logistic regression model was of good fit. However, the classification table showed 74% overall correct prediction of those who sought care within 3 days. According to the Nagelkerke test, the independent variables can explain 14.1% of the variation in the dependent variables.

## Discussion

As shown in this study, the majority of the survivors seeking care, 70%, did so within the three-day window that would enable them to receive the full package of care, but this varied greatly between the two study sites. Most survivors sought care at the clinics specialised in sexual violence (Tumaini clinics) and this was true for both female and males and for all age groups. Further, among survivors seeking care, the proportion of those who were males or children was low. In addition, we show that male survivors were significantly more likely than female survivors to seek care within 3 days whereas survivors in all age groups under 50 years were significantly more likely to seek care within 3 days compared to those aged 50 years and older. Survivors being referred by the community, family member, mobile clinic or local authorities were less likely to seek care within 3 days, compared to survivors who were self-referred.

The low proportion of male survivors accessing care among the total number of survivors in our findings is in line with one previous study of survivors accessing care in North Kivu [20]. Prevalence numbers obtained from the study area, showing that 39.7% of women and 23.6% of men had experienced sexual violence [5], indicate a ratio of 0.6 male per 1 female survivor, which leads us to speculate that male survivors are less likely to access care compared to female survivors in this area. This might have several explanations such as stigmatisation, male gender roles, and self-blaming [29,30], awareness raising activities focusing more on female survivors, the way in which care is organised and lack of confidence among staff to identify male survivors. Even though women and girls are disproportionally affected by sexual violence, the access to care for male survivors also needs attention and can be addressed by adapting services to be more acceptable to and accessible by males for example, by integrating sexual violence services with other less stigmatising services, including information about male survivors in awareness raising activities and, improving identification of male survivors. More research is needed to further explore how male survivors prefer to access and receive care.

The broad age range shown in our findings, 1–80 years, is in line with previous studies of survivors in eastern DRC [3,20] and indicates that sexual violence can happen to anyone, regardless of age. The age group most commonly accessing care was 20–29 years old, which is also coungruent with previous studies [19,20] from DRC, even though one of the studies found younger survivors more commonly accessing care in one of their two study areas [20]. Self-referral was the most common referral pathway for this age group, which might indicate that this age group has the best access to information, either through awareness raising activities or by peers. Our findings show that 26% of the survivors accessing care were adolescents (10–19 years) and 2% were under 10 years old. Even though child sexual abuse has been reported as common in DRC [31], other studies of survivors accessing care also show low proportions of children, ranging from 0% of survivors being under 10 years old and between 1% and 16% of survivors being under 16 years old [3,19,32]. It is reasonable to assume that not all child survivors are accessing care, considering that children do not always have the words or the ability to recognise and describe the abuse. It has been shown that child sexual abuse in DRC is much more likely to be perpetrated by a civilian and someone known to the survivor, compared to adult sexual violence [31,33], which might make it more difficult to disclose the incident. This could be addressed through more awareness raising activities around child sexual abuse, adapting services to be more child friendly and training staff to be aware of signs and symptoms of child sexual abuse. The finding that survivors in the lower age groups were more likely to seek care within 3 days compared to survivors aged 50 years and older is consistent with the results of other studies from DRC showing that child survivors are more likely to seek care early [3,33]. This indicates that children are overall less likely to access care but when they do, they tend to seek care early.

The proportion of survivors seeking care within 3 days varies among different studies from the surrounding areas. Most studies from DRC report lower proportions of survivors accessing care within 72 hours than those found in this study, varying from 0.6% to 46% [2,3,13,19]. One study from another MSF programme in DRC showed proportions similar to ours from one of their research sites (60%) but lower proportions from the second study location (32%) [20] and a study from South Kivu showed a high proportion of survivors accessing care within 72 hours in the healthcare centres (86%) but lower proportion (12%) in the hospital [21]. The MSF awareness raising activities in the area, especially in Mweso, have been intense in promoting the importance of seeking care within 3 days, which could have had a positive impact on knowledge about the urgency of seeking care among the population and explain the difference in timing between the two study locations. It is however important to look at this indicator critically. If the community perception is that one has to come within 3 days, there is a risk that survivors who cannot access care within 3 days will not present for health care at all. Even if it is important for survivors to access care within 3 days in order to be able to prevent HIV transmission, it is also important that survivors who for different reasons cannot access care within 3 days still access care whenever it is possible and this needs to be well communicated in awareness raising activities. Timely access to care was higher in the health centres compared to the Tumainis, which might be explained by the physical proximity to the health centres that are more densely situated in the rural areas, but might also be a result of less knowledge about the possibility to seek care after 3 days in the more remote areas. This would need further investigation to further the understanding.

Male survivors were more likely to seek care within 3 days after the sexual violence incident compared to female survivors, which might be related to the fact that women in DRC (especially in rural areas) to a greater extent than men lack power and income [34]. Another reason might be that the few male survivors who accessed care were the ones who were well informed and courageous enough to seek care and therefore also more likely to be aware of the need of timely care and having the resources to access care in time.

Most survivors were self-referred, which is congruent with another study from North Kivu [20]. However, one study from South Kivu showed that other NGOs are the most common referral pathway [3]. That was not observed in our findings but can be explained by the fact that there are very few other providers offering support services to survivors of sexual violence in the two study locations. Mapping and collaboration with other service providers as well as collaboration with local authorities for set-up of referral systems are crucial for a successful sexual violence intervention and this needs to be strengthened within the study area.

Being referred from the community, by a family member, mobile clinic or local authorities was associated with lower odds of seeking care within 3 days. This finding should be interpreted cautiously since it is not known at what moment the survivor accessed the respective instance for referral. The finding might, however, indicate that the referring instances lack information about the medical emergency of a sexual violence incident and thus do not refer the survivor immediately, but it might indicate that survivors disclosing late tend to do so to one of those instances instead of seeking care directly. More research focusing on help- and care-seeking behaviours and first point of contact for survivors as well as assessment of knowledge and willingness to refer among community members, local authorities and other service providers is needed to further understand this finding and to improve timely referrals.

Most survivors in this study sought care at one of the 3 Tumaini clinics. This was the case for female and male survivors within all age groups. Reasons for this might be that all the Tumaini clinics are located in larger villages, facilitating access from several major roads. The services provided in specialised facilities are likely to be well known and well perceived by the population, since the staff are specialised in sexual violence and the facilities are mentioned in awareness raising activities in the areas. The professional attitudes among the staff might also be more appropriate within a specialised clinic. We also show that the self-referral and referral from a health promoter was common in the Tumaini clinics, whereas referrals from local authorities were more common in the hospitals. This indicates that the specialised services are widely known among the health promoters and general population but either not well known or not preferred by authorities. This needs further exploration, as well as adapted actions to ensure survivors referred through authorities also have the opportunity to choose the healthcare facility of their preference.

There are potential negative effects of specialised clinics, e.g. increased stigmatisation of a survivor visiting a Tumaini clinic due to the nature of the clinic, even if other services are provided to decrease the risk of exposure. However, as survivors seemed to prefer to access care at the specialised clinics in both study locations, this model of care should be further studied and more widely implemented.

### Methodological considerations

The major limitation of the study is that only survivors of sexual violence who accessed health care within MSF programmes were included. Thus, nothing is known about the demographic background and help-seeking behaviours of survivors not accessing care at all or accessing care from other providers. This limitation must be kept in mind when interpreting the results

This study uses data from medical records which were collected before the study design was developed. Information about other factors that might influence timely access to care, such as socio-economic status, education level, type of perpetrator, whether the violence was conflict-related and distance to healthcare facility was not available.

The accounts of sexual violence incidents in the study were based on self-report by survivors seeking and receiving care after sexual violence. It is possible that some patients presenting as survivors were presenting with incorrect claims, for example in order to receive free health care. However, given the high level of stigma and negative social consequences for survivors of sexual violence in the area and that primary health care including sexual and reproductive health services were free for all patients within the same healthcare facilities or nearby facilities, we believe that such false accounts were rare.

The day of the incident was based on self-report and could thus be subject to recall bias. In addition, the data did not contain any information about the exact time for the sexual violence incident nor the consultation, only the dates. Survivors arriving on day 0–3 after the incident were counted as having accessed care within 3 days, which might include some survivors seeking care up to 96 hours after the incident.

The data used for the study were obtained only from two MSF programme areas in different parts of North Kivu. It is known that care seeking behaviours vary across different contexts and different cultures; thus, the findings from this study might have limited generalisability.

The study includes data from 5 years, which provides a large sample size. However, the context in the study setting has been changing over these 5 years as has the MSF support. While the use of data covering a shorter time period might have provided a more stable context, the added value of a larger sample size was considered greater.

## Conclusion

Only a small proportion of sexual violence survivors accessing care in the two MSF programmes in North Kivu are male or children and access to timely care remains a challenge. However, this study does not address the underlying factors affecting the access to care. Additional qualitative studies are needed to further understand the underlying reasons for the care-seeking patterns shown in this study and to further explore how timely access to care for survivors of sexual violence can be improved.

To overcome the barriers to seeking health care, models of care should be developed in close collaboration with communities to ensure that their needs are being met. The set-up of healthcare services needs to be welcoming and accessible for all survivors. Awareness raising activities need to target different groups and include messages about different types of sexual violence as well as different types of survivors. Specialised clinics, like the Tumaini clinics, can be one model to further implement, as well as integrating the specialised clinics into already existing healthcare facilities. In addition to the set-up of the healthcare services and awareness raising activities there is also need for well-established referral pathways with support services available and coordination and collaboration between actors offering support to survivors of sexual violence.

## Paper context

Timely access to care for survivors of sexual violence is crucial yet challenging in many places, including in North Kivu, DRC. This study shows that a majority of survivors access care through specialised clinics, that access is limited for male and child survivors and highlights factors influencing timely access to care for survivors of sexual violence. This information can be used to adapt programmes and improve access to timely care for survivors of sexual violence.
